# Synthesis and Characterization of a New Bivalent Ligand Combining Caffeine and Docosahexaenoic Acid

**DOI:** 10.3390/molecules22030366

**Published:** 2017-02-27

**Authors:** Víctor Fernández-Dueñas, Jhonny Azuaje, Xavier Morató, Begoña Cordobilla, Joan Carles Domingo, Eddy Sotelo, Francisco Ciruela

**Affiliations:** 1 Unitat de Farmacologia, Departament Patologia i Terapèutica Experimental, Facultat de Medicina, IDIBELL-Universitat de Barcelona, L’Hospitalet de Llobregat, 08907 Barcelona, Spain; vfernandez@ub.edu (V.F.-D.); xmorato_16@hotmail.com (X.M.); 2 Institut de Neurociències, Universitat de Barcelona, 08035 Barcelona, Spain; 3 Centro Singular de Investigación en Química Biolóxica e Materiais Moleculares (CIQUS), Universidad de Santiago de Compostela, 15782 Santiago de Compostela, Spain; j.azuaje@usc.es; 4 Departamento de Química Orgánica, Facultad de Farmacia, Universidade de Santiago de Compostela, Santiago de Compostela, 15782 Santiago de Compostela, Spain; 5 Departament de Bioquímica i Biomedicina, Universitat de Barcelona, 08028 Barcelona, Spain; bgcordobilla07@ub.edu (B.C.); jcdomingo@ub.edu (J.C.D.)

**Keywords:** adenosine A_2A_ receptor, caffeine, docosahexaenoic acid (DHA), inverse agonism

## Abstract

Caffeine is a promising drug for the management of neurodegenerative diseases such as Parkinson’s disease (PD), demonstrating neuroprotective properties that have been attributed to its interaction with the basal ganglia adenosine A_2A_ receptor (A_2A_R). However, the doses needed to exert these neuroprotective effects may be too high. Thus, it is important to design novel approaches that selectively deliver this natural compound to the desired target. Docosahexaenoic acid (DHA) is the major omega-3 fatty acid in the brain and can act as a specific carrier of caffeine. Furthermore, DHA displays properties that may lead to its use as a neuroprotective agent. In the present study, we constructed a novel bivalent ligand covalently linking caffeine and DHA and assessed its pharmacological activity and safety profile in a simple cellular model. Interestingly, the new bivalent ligand presented higher potency as an A_2A_R inverse agonist than caffeine alone. We also determined the range of concentrations inducing toxicity both in a heterologous system and in primary striatal cultures. The novel strategy presented here of attaching DHA to caffeine may enable increased effects of the drug at desired sites, which could be of interest for the treatment of PD.

## 1. Introduction

Caffeine, the most consumed psychoactive substance worldwide, is generally used because it enhances physical and cognitive functions, improving alertness, physical performance and concentration, while decreasing fatigue [1,2]. Caffeine is also used as an adjuvant drug in pain relief [3]. In addition, it has been postulated that this natural substance may have beneficial effects in neurodegenerative diseases such as Parkinson’s disease (PD) [4]. Indeed, several epidemiological studies have revealed an inverse relationship between caffeine consumption and the risk of developing PD [5,6,7], suggesting that caffeine can act as a neuroprotective agent [8,9]. The mechanism of action of caffeine, at non-toxic concentrations, involves interacting with and blocking adenosine receptors [10]. We recently reported that caffeine may act as an inverse agonist for adenosine A_2A_ receptors (A_2A_R) under certain circumstances [11], suppressing constitutive A_2A_R activity. Interestingly, A_2A_R may be over-expressed in PD, leading to increased activity within the basal ganglia [12,13], which could explain the neuroprotective effects exerted by the inverse agonist activity of caffeine. However, the potency of caffeine is relatively low and high doses are needed to achieve significant effects (i.e., neuroprotective and motor effects) [14,15]. Hence, it is important to discover how to increase caffeine levels at specific sites to promote its therapeutic effects.

One of the current approaches in drug delivery consists of using lipid carriers that help drugs reach their target [16,17]. One of these carriers is docosahexaenoic acid (DHA), which displays special characteristics that may potentiate its use. DHA is the major omega-3 fatty acid present in the mammalian brain, where it is involved in a number of essential processes such as neurogenesis and neurotransmission. Esterified DHA is found in the plasma membrane phospholipid bilayer, where it contributes to the maintenance of its physical properties, including membrane organization, ion permeability, elasticity and microdomain formation [18,19]. We recently showed that A_2A_R and dopamine D_2_ receptor (D_2_R) expression is increased in DHA-enriched microdomains, eliciting an increased rate of receptor oligomerization in the striatum [20]. Therefore, the rationale for attaching caffeine to DHA (caffeine-DHA) is primarily to target the delivery of caffeine to over-expressed striatal A_2A_Rs. In addition to carrying caffeine to A_2A_R-containing membranes, DHA may also promote its retention in the target tissue, increasing the interaction time between caffeine and A_2A_R. Furthermore, DHA has been considered a neuroprotective or even a disease-modifying agent in the treatment of PD [21]. DHA is not only considered important for optimal cognitive health and neuronal development [22], but it has also been shown to exert neuroprotective effects in a number of studies [23]. Hence, a possible synergy between caffeine and DHA might provide additional neuroprotection, which may lead to better management of neurodegenerative conditions.

In the present study, we aimed to design and synthesize a new bivalent ligand containing caffeine and DHA (caffeine-DHA), and to characterize its pharmacological activity and possible toxicity in a simple cellular model.

## 2. Results and Discussion

**Figure 1:**
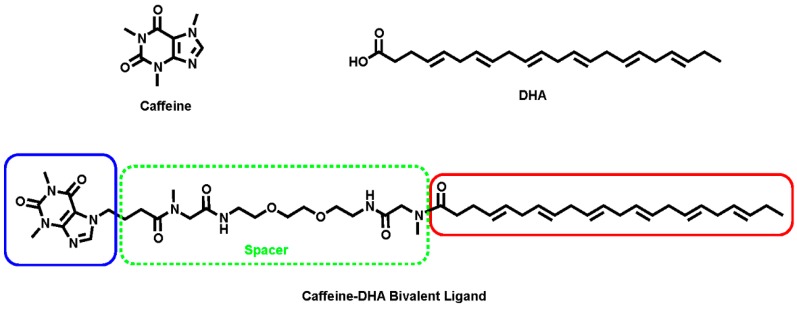
Structure of caffeine, docosahexaenoic acid (DHA) and the caffeine-DHA bivalent ligand.

**Figure 2:**
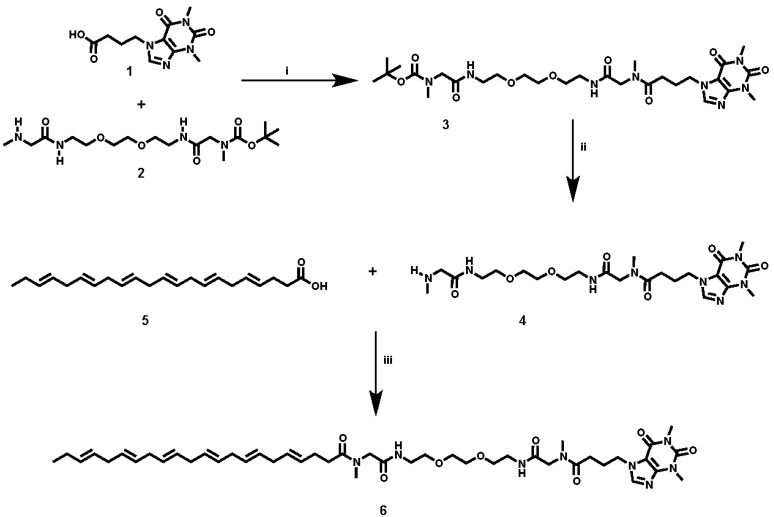
Synthesis of the caffeine-DHA hybrid structure (**6**). (**i**) Synthesis of *tert*-butyl (19-(1,3-dimethyl-2,6-dioxo-2,3-dihydro-1*H*-purin-7(6*H*)-yl)-15-methyl-2,13,16-trioxo-6,9-dioxa-3,12,15-triazanonadecyl)(methyl)carbamate (**3**); (**ii**) Synthesis of 4-(1,3-dimethyl-2,6-dioxo-2,3-dihydro-1*H*-purin-7(6*H*)-yl)-*N*-(4,15-dioxo-8,11-dioxa-2,5,14-triazahexadecan-16-yl)-*N*-methylbutanamide (**4**); (**iii**) Synthesis of (4*E*,7*E*,10*E*,13*E*,16*E*,19*E*)-*N*-(19-(1,3-dimethyl-2,6-dioxo-2,3-dihydro-1*H*-purin-7(6*H*)-yl)-15-methyl-2,13,16-trioxo-6,9-dioxa-3,12,15-triazanonadecyl)-*N*-methyldo-cosa-4,7,10,13,16,19-hexaenamide (**6**).

Caffeine and DHA were used to synthesize the caffeine-DHA bivalent ligand (Figure 1), using the synthetic procedure presented in Figure 2. Briefly, reaction of the acid-functionalized caffeine precursor (**1**) with *tert*-butyl (4,15-dioxo-8,11-dioxa-2,5,14-triazahexa-decan-16-yl)(methyl)carbamate (**2**), using 1-Ethyl-3-(3-dimethylaminopropyl)carbodiimide (EDC) as a coupling reagent, enabled the introduction of the selected spacer and linker groups into the caffeine scaffold, thereby yielding the caffeine derivative (**3**). Cleavage of the Boc group at (**3**), by treatment with TFA at 0 °C, and the subsequent coupling of (**4**) with DHA (**5**) under typical coupling conditions (EDC) provided a rapid way of generating the caffeine-DHA hybrid (**6**).

**Figure 3:**
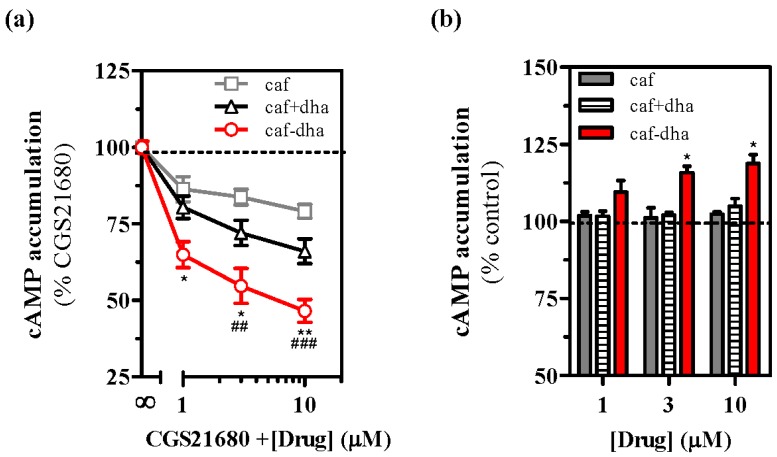
Evaluation of caffeine-DHA intrinsic activity. (**a**) cAMP measurements in A_2A_R-expressing cells incubated with a fixed concentration of CGS21680 (200 nM) in the absence/presence of increasing concentrations of caffeine (gray squares), caffeine plus DHA (black triangles) and caffeine-DHA (red circles). cAMP levels obtained by the CGS21680 concentration were set as 100%. Data represent the mean ± s.e.m. of four independent experiments. Asterisks (when comparing caffeine plus DHA and caffeine-DHA treatments) or hashtags (when comparing caffeine and caffeine-DHA treatments) indicate significant differences between drug treatments for each concentration (* *p* < 0.05, ** or ^##^ *p* < 0.01 and ^###^ *p* < 0.001, one-way ANOVA followed by Bonferroni’s post hoc test); (**b**) cAMP measurements in A_2A_R-transfected cells following incubation with caffeine, caffeine plus DHA or caffeine-DHA. Basal cAMP levels (control) were set as 100%. Data represent the mean ± s.e.m. of four independent experiments. Asterisks indicate significant differences between drug treatments for each concentration (* *p* < 0.05, one-way ANOVA followed by Bonferroni’s post hoc test).

Drug development is based on characterizing two main aspects of the novel molecules, the pharmacological activity and the possible toxicity. Here, we performed in vitro screening to examine the intrinsic activity and toxicity of caffeine-DHA. First, we evaluated the effects of the novel drug on A_2A_R activity by measuring cAMP accumulation, which results from A_2A_R-dependent canonical intracellular signaling [24]. The effects of caffeine-DHA on cAMP production induced by CGS21680 (an A_2A_R selective agonist) in cells expressing A_2A_R were evaluated. At 200 nM, CGS21680 induced an increase in cAMP levels of ~75% compared to the control (forskolin-induced cAMP accumulation at 100%). Caffeine-DHA dose-dependently inhibited CGS21680-mediated cAMP accumulation (Figure 3a), confirming that the novel drug could bind to A_2A_R. In addition, the efficacy of caffeine-DHA in suppressing cAMP accumulation was greater than that of caffeine administered alone or with DHA at equimolar concentrations. The efficacy of caffeine was not significantly affected when not covalently bound to DHA, indicating that being covalently bound to DHA had a major effect on caffeine potency. To evaluate the effect of caffeine-DHA on basal cell signaling, we administered increasing concentrations of the drug in the absence of an A_2A_R-selective agonist. Caffeine-DHA increased cAMP levels (Figure 3b), while caffeine alone or co-administered with DHA had no effect. Of note, neither caffeine alone nor caffeine with DHA affected cAMP production in mock cells not expressing A_2A_R (data not shown). It should also be noted that caffeine at higher concentrations also elevated cAMP levels (data not shown), as previously described [11], thus indicating that both caffeine-DHA and caffeine are inverse agonists of A_2A_R, with caffeine-DHA exhibiting a higher potency than caffeine alone.

According to the abovementioned data, caffeine-DHA displayed promising pharmacological activity, validating our approach of attaching a DHA molecule to caffeine to increase affinity/activity for cognate receptors. In recent years, the use of lipid-based nanoparticles as drug carriers has been a common approach in pharmaceutics (for review, see [16]). Similarly, the use of antibody-drug or small molecule–drug conjugates to specifically target the desired tissue has become increasingly important in cancer therapy [25]. Here, we did not aim to develop a selective carrier for caffeine, but to increase the availability of caffeine in A_2A_R-containing sites, which would need to be properly assessed (i.e., by radioligand binding assays) in further studies. This strategy is not new, since a number of lipid-based carriers (i.e., fatty acids, glycerides and phospholipids) have been previously used to design lipid prodrugs (for review, see [26]). We therefore designed our drug based on the new concepts emerging in pharmacology, such as the residence time [27] to facilitate the drug-receptor interaction. According to our data, caffeine-DHA exhibited a high capacity for interacting with A_2A_R, which may be due to the insertion of the DHA domain into the plasma membrane. Moreover, an intrinsic effect of DHA could also explain, at least in part, the results obtained, given that the co-administration of unmodified caffeine with DHA also produced some effects, although they were not significant compared to those of caffeine alone when competing with CGS21680. Recently, an effect of DHA on receptor complex availability in the plasma membrane was reported [20], which could enhance the effect of the drug being delivered. Finally, another aspect to be taken into account is the introduction of a spacer between the caffeine and DHA molecules. We designed this spacer to enable the insertion of DHA into the plasma membrane while allowing the caffeine molecule to interact with A_2A_R. However, the resulting ligand had a very high molecular weight, which could result in pharmacokinetic problems (i.e., distribution and the route of administration) when applied therapeutically. Thus, the length of the spacer might need to be explored further to obtain the best compromise between pharmacodynamic and pharmacokinetic properties.

**Figure 4:**
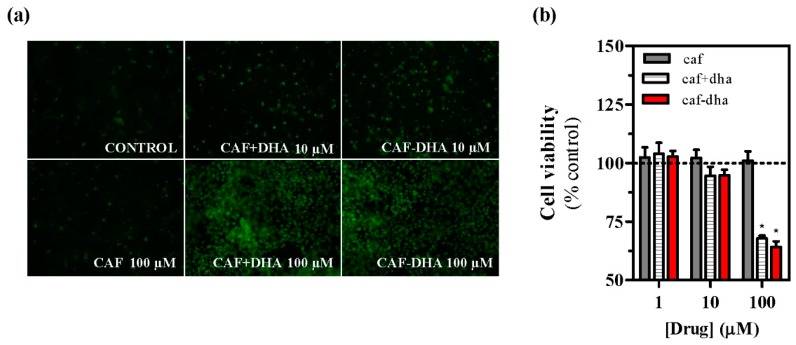
Evaluation of caffeine-DHA toxicity. (**a**) Representative images of A_2A_R-expressing cells incubated with either HBSS (control) or the drug (caffeine, caffeine plus DHA or caffeine-DHA) and subsequently treated with propidium iodide (10 µM). The drug and concentration are indicated in each image; (**b**) Fluorescence levels obtained in A_2A_R-transfected cells treated with propidium iodide (10 µM) after drug (caffeine, caffeine plus DHA or caffeine-DHA) incubation. Data represent the mean ± s.e.m. of four independent experiments. Basal viability levels (control) were set as 100%. Asterisks indicate significant differences between drug treatments for each concentration (* *p* < 0.05, one-way ANOVA followed by Bonferroni’s post hoc test).

**Figure 5:**
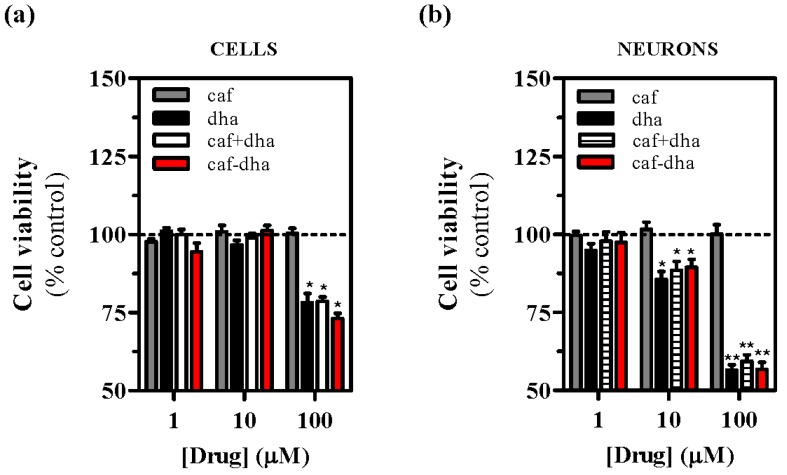
Evaluation of caffeine-DHA toxicity and neuroprotection. (**a**) Absorbance levels obtained in A_2A_R-expressing cells incubated with either HBSS (control) or the drug (caffeine, DHA, caffeine plus DHA or caffeine-DHA) and subsequently treated with MTT (0.5 mg/mL); (**b**) Absorbance levels obtained in striatal neurons treated with MTT (0.5 mg/mL) after drug (caffeine, DHA, caffeine plus DHA or caffeine-DHA) incubation. Data represent the mean ± s.e.m. of four independent experiments. Basal viability levels (vehicle) were set as 100%. Asterisks indicate significant differences between drug treatments for each concentration (* *p* < 0.05 and ** *p* < 0.01, one-way ANOVA followed by Bonferroni’s post hoc test).

The safety profile of the novel drug was assessed by measuring cell viability after treatment, using the well-characterized agent propidium iodide, which is not able to insert into the DNA of intact living cells [28]. A_2A_R-expressing cells incubated with caffeine-DHA at concentrations higher than 10 µM showed high mortality (Figure 4a,b). While caffeine did not induce toxicity at any concentration, caffeine-DHA had a dramatic effect on cell viability at higher concentrations (Figure 4a,b). Interestingly, the co-administration of caffeine and DHA also produced cell toxicity, suggesting that DHA could be responsible for the observed cell death. A possible explanation for this toxic effect could be that high concentrations of DHA destabilize the plasma membrane, an event that would not occur physiologically as the tissue would buffer any lipid excess. This concentration-dependent toxicity of caffeine-DHA was further confirmed by MTT viability assays and in neuronal cell cultures. Again, high concentrations (100 µM) of caffeine-DHA induced cell death, as did the administration of DHA alone or with caffeine (Figure 5a). Furthermore, primary striatal cultures were more susceptible to toxicity than HEK293T cells, since cell survival of the primary cultures was significantly affected even at intermediate concentrations (10 µM) (Figure 5b). Thus, caffeine-DHA exhibited adverse effects when used at high concentrations, but was effective and safer at low concentrations. Although adverse effects are a major issue in drug development, we showed that caffeine-DHA can be used safely, albeit in a small range of concentrations. However, dosing in whole organisms is still a challenge and requires a benefit/risk profile. In fact, DHA has been shown to be safe in a number of studies and is a dietary supplement for a number of pathologies, including PD [21]. Hence, it is reasonable to continue developing caffeine-DHA in both pre-clinical and clinical studies.

## 3. Materials and Methods

### 3.1. Drugs and Reagents

The reagents used were forskolin from Sigma-Aldrich (St. Louis, MO, USA), and CGS21680 and caffeine from Tocris Bioscience (Ellisville, MO, USA). Adenosine deaminase was purchased from Roche Diagnostics GmbH (Mannheim, Germany) and zardaverine from Calbiochem (San Diego, CA, USA). Docosahexaenoic acid in the free form was a generous gift from Brudy Technology (Barcelona, Spain), containing more than 70% of DHA in total fatty acids (omega-3 fatty acid content over 95%).

### 3.2. Synthesis of Caffeine-DHA

#### 3.2.1. Chemistry 

Unless otherwise indicated, all starting materials, reagents and solvents were purchased and used without further purification. After extraction from the aqueous phases, the organic solvents were dried over anhydrous Na_2_SO_4_. The reactions were monitored by thin-layer chromatography (TLC) on 2.5 mm Merck (Kenilworth, NJ, USA) silica gel GF 254 strips, and the purified compounds each showed a single spot. Unless stated otherwise, UV light and/or iodine vapor were used to detect compounds. Most of the preparative experiments were performed in coated vials on an organic synthesizer with orbital stirring. The purity and identity of all the tested compounds were established by a combination of HPLC, elemental analysis, mass spectrometry and NMR spectroscopy, as described below. Purification of isolated products was carried out by column chromatography (Kieselgel 0.040–0.063 mm, E. Merck, Darmstadt, Germany) or medium pressure liquid chromatography (MPLC) on a CombiFlash Companion (Teledyne ISCO, St, Lincoln, NE, USA) with RediSep pre-packed normal-phase silica gel columns (35–60 µm), followed by recrystallization. Melting points were determined on a Gallenkamp melting point apparatus and were uncorrected. The NMR spectra were recorded on Bruker AM300 and XM500 spectrometers (Bruker AXS Inc., Madison, WI, USA). Chemical shifts are given as δ values against the internal standard tetramethylsilane and *J* values are given in Hz. Mass spectra were obtained on a Varian MAT-711 instrument (Agilent Technologies Inc., Santa Clara, CA, USA). High-resolution mass spectra were obtained on an AutoSpec Micromass spectrometer (Micromass Inc., Cary, NC, USA). Analytical HPLC was performed on an Agilent 1100 system (Agilent Technologies Inc., Santa Clara, CA, USA) using an Agilent Zorbax SB-Phenyl, 2.1 mm × 150 mm, 5 µm column with gradient elution using the mobile phases (A) H_2_O containing 0.1% CF_3_COOH and (B) MeCN at a flow rate of 1 mL/min. The purity of all the tested compounds was >95%.

#### 3.2.2. Synthesis of *tert*-Butyl (19-(1,3-dimethyl-2,6-dioxo-2,3-dihydro-1*H*-purin-7(6*H*)-yl)-15-methyl-2,13,16-trioxo-6,9-dioxa-3,12,15-triazanonadecyl)(methyl)carbamate (**3**)

To a mixture of the acid-functionalized caffeine precursor (**1**) (133 mg, 0.5 mmol), the *N*-Boc-protected sarcosine-amide derivative (**2**) (292 mg, 0.75 mmol) and DMAP (67 mg, 0.55 mmol) in DCM (15 mL) were added in *N*-[(dimethylamino)propyl]-*N*’-ethylcarbodiimide hydrochloride (115 mg, 0.6 mmol) at room temperature. The reaction mixture was stirred overnight at room temperature, concentrated and the residue dissolved in ethyl acetate and washed with water. The ethyl acetate layer was dried over sodium sulfate and concentrated to give the crude product, which was chromatographed on silica gel using DCM/MeOH to yield 229 mg (72%) of the product as a white solid. LCMS purity was 98.7% and m.p. 195–196 °C. ^1^H-NMR (300 MHz, CDCl_3_) δ (ppm): 7.33 (t, *J* = 7.5 Hz, 1H), 7.18 (s, 1H), 6.75 (t, *J* = 7.4 Hz, 1H), 4.20–4.09 (m, 2H), 3.98 (s, 2H), 3.88 (s, 2H), 3.67–3.47 (m, 10H), 3.46–3.36 (m, 2H), 3.02 (s, 3H), 2.96 (s, 3H), 2.86 (s, 6H), 2.37 (t, *J* = 6.0 Hz, 2H), 2.09–1.93 (m, 2H), and 1.27 (s, 9H). HRMS (CI) *m/z* calculated for C_28_H_47_N_8_O_9_ [M + H]^+^ was 639.3463 and found to be 639.3468.

#### 3.2.3. Synthesis of 4-(1,3-Dimethyl-2,6-dioxo-2,3-dihydro-1*H*-purin-7(6*H*)-yl)-*N*-(4,15-dioxo-8,11-dioxa-2,5,14-triazahexadecan-16-yl)-*N*-methylbutanamide (**4**)

TFA (0.25 mL, 3.3 mmol) was added to a solution of the caffeine derivative (**3**) (200 mg, 0.3 mmol) in dichloromethane (15 mL) at 0 °C. The reaction mixture was removed from the ice bath and stirred at room temperature for 3 h. The solvents were removed in vacuo and the residue diluted with dichloromethane. The mixture was neutralized with saturated aqueous sodium bicarbonate. The organic layer was separated, dried (sodium sulfate) and concentrated in vacuo to provide the crude product, which was chromatographed on silica gel using DCM/MeOH to yield 136 mg (81%) of the product as a white solid. LCMS purity was 98.4% and m.p. 214–215 °C. ^1^H-NMR (300 MHz, CDCl_3_) δ (ppm): 7.38 (t, *J* = 7.6 Hz, 1H), 7.21 (s, 1H), 6.70 (t, *J* = 7.6 Hz, 1H), 4.15–4.04 (m, 2H), 3.95 (s, 2H), 3.81–3.72 (m, 2H), 3.61–3.42 (m, 10H), 3.39–3.19 (m, 3H), 3.04 (s, 3H), 2.87 (s, 6H), 2.67 (s, 3H), 2.41 (t, *J* = 6.2 Hz, 2H), and 2.10–1.94 (m, 2H). HRMS (CI) *m*/*z* calculated for C_23_H_39_N_8_O_7_ [M + H]^+^ was 539.2938 and found to be 539.2942.

#### 3.2.4. Synthesis of (4*E*,7*E*,10*E*,13*E*,16*E*,19*E*)-*N*-(19-(1,3-Dimethyl-2,6-dioxo-2,3-dihydro-1*H*-purin-7(6*H*)-yl)-15-methyl-2,13,16-trioxo-6,9-dioxa-3,12,15-triazanonadecyl)-*N*-methyldo-cosa-4,7,10,13,16,19-hexaenamide (**6**)

To a mixture of DHA (**5**) (50 mg, 0.15 mmol), the sarcosinamide-functionalized caffeine precursor (**4**) (100 mg, 0.187 mmol) and DMAP (20 mg, 0.165 mmol) in DCM (20 mL) were added in *N*-[(dimethylamino)propyl]-*N*’-ethylcarbodiimide hydrochloride (35 mg, 0.18 mmol) at room temperature. The reaction mixture was stirred overnight at room temperature, concentrated and the residue dissolved in ethyl acetate and washed with water. The ethyl acetate layer was dried over sodium sulfate and concentrated to give the crude product, which was chromatographed on silica gel using EtOAc/MeOH to give 57 mg (45%) of the product as a pale yellow oil. LCMS purity was 97.2%. ^1^H-NMR (300 MHz, CDCl_3_) δ (ppm): 7.21 (s, 1H), 7.00 (brs, 1H), 6.82 (brs, 1H), 5.47–5.22 (m, 12H), 4.27–4.15 (m, 2H), 4.02 (s, 2H), 3.99 (s, 2H), 3.61–3.36 (m, 12H), 3.09 (s, 3H), 3.05 (s, 3H), 2.91 (s, 3H), 2.85 (s, 3H), 2.48–2.26 (m, 10H), 2.19–1.92 (m, 10H), and 0.96 (t, *J* = 7.5 Hz, 3H). HRMS (ESI) *m*/*z* calculated for C_45_H_69_N_8_O_8_ [M + H]^+^ was 849.5236 and found to be 849.5244.

### 3.3. Plasmids and Transfection

The cDNA encoding the A_2A_R^SNAP^ [29] was used. Human embryonic kidney (HEK)293T cells were grown at 37 °C, 5% CO_2_, in Dulbecco’s modified Eagle’s medium (DMEM) (Sigma-Aldrich, Saint Louis, MO, USA) supplemented with 1 mM sodium pyruvate, 2 mM l-glutamine, 100 U/mL streptomycin, 100 mg/mL penicillin and 5% (*v*/*v*) fetal bovine serum. Cells were seeded on to six-well plates at 300,000 cells/well and transiently transfected using Transfectin (Bio-Rad Laboratories, Hercules, CA, USA), following the manufacturer’s instructions.

### 3.4. Animals and Neuronal Cultures

CD-1 mice (from the animal facility of the University of Barcelona) weighing 20–25 g were used. The University of Barcelona’s Committee on Animal Use and Care approved the protocol. Animals were housed and tested in compliance with the guidelines described in the Guide for the Care and Use of Laboratory Animals [30] and followed European Union directives (2010/63/EU). All efforts were made to minimize animal suffering and the number of animals used. All animals were housed in groups of five in standard cages with ad libitum access to food and water, and maintained under a 12 h dark/light cycle (starting at 7:30 a.m.) and at 22 °C and 66% humidity (standard conditions).

Primary striatal neurons were cultured from E18 CD-1 mouse embryos. Briefly, after dissection, the striatum was treated with 1.25% trypsin (Sigma-Aldrich, St. Louis, MO, USA) for 10 min and mechanically dissociated with a flame-polished Pasteur pipette. Neurons were plated onto poly-d-lysine–coated (0.1 mg/mL) and laminin-coated (0.01 mg/mL) wells at a density of 80,000 cells/cm^2^ on a six-well plate for cAMP accumulation assays in minimum essential medium (Invitrogen, Carlsbad, CA, USA) supplemented with 10% horse serum, 10% bovine serum, 1 mM pyruvic acid and 0.59% glucose. After 4–14 h, the medium was substituted with Neurobasal medium supplemented with penicillin (100 U/mL), streptomycin (100 μg/mL), 0.59% glucose and B27 supplement (Invitrogen Carlsbad, CA, USA). Neurons were kept at 5% CO_2_, 37 °C and 95% humidity for 21 days before the experiments.

### 3.5. cAMP Accumulation Assay

cAMP accumulation was measured using the LANCE^®^ *Ultra* cAMP kit (PerkinElmer, Waltham, MA, USA), as previously described [31]. Briefly, cells were incubated for 1 h at 37 °C with stimulation buffer (0.1% bovine serum albumin (BSA), 0.5 units/mL of adenosine deaminase and 2 µM zardaverine in serum-free DMEM) and later with the different drugs for 30 min at 37 °C. Thereafter, cells were transferred to a 384-well plate in which reagents were added following the manufacturer’s instructions. After 1 h at room temperature, TR-FRET was determined by measuring light emission at 620 nm and 665 nm using a POLARstar plate reader (BMG Labtech, Durham, NC, USA).

### 3.6. Propidium Iodide Assay

Cell viability was evaluated by assessing the amount of propidium iodide penetrating living cells, as previously described [32]. Briefly, A_2A_R-expressing cells treated with the drug for 3 h were incubated with propidium iodide (Sigma-Aldrich; 10 µM) for 30 min at 37 °C. After two wash steps with Hank’s Balanced Salt Solution (HBSS; containing, in mM, 137 NaCl, 0.34 Na_2_HPO_4_, 5 KCl, 0.44 KH_2_PO_4_, 0.5 MgCl_2_, 0.4 Mg_2_SO_4_, 1.26 CaCl_2_, 10 HEPES, 2 d-glucose and 1 ascorbic acid; adjusted to pH 7.4 with NaOH), light emission at 630 nm was measured after excitation at 495 nm in a POLARstar plate reader or the cells were visualized in a FLoid Cell Imaging Station (Life Technologies, Carlsbad, CA, USA).

### 3.7. MTT Assay

Drug toxicity was evaluated by measuring the cellular uptake of 3-(4,5-dumethylthiazol-2-yl)-2,5-iphenyltetrazolium bromide (MTT), as previously described [33]. Briefly, A_2A_R-expressing cells treated with the drug for 3 h were incubated with a solution of MTT (Sigma-Aldrich; 0.5 mg/mL) in HBSS for 30 min at 37 °C. The tetrazolium ring of MTT is cleaved by active dehydrogenases in living cells to produce a precipitated formazan salt that is solubilized by DMSO to give a colored compound. After stirring the mixture for 5 min, optical density was measured at 540 nm in the POLARstar plate reader to determine the rates of cell death.

### 3.8. Statistical Analysis

The number of samples (n) in each experimental condition is indicated in the figure legends. Statistical analysis was performed by one-way ANOVA followed by Bonferroni’s multiple comparison post hoc test. Statistical significance is indicated for each experiment.

## 4. Conclusions

We have synthesized a novel drug by combining caffeine and DHA using a simple and efficient synthetic procedure. This ligand, caffeine-DHA, is a more potent A_2A_R inverse agonist than caffeine alone. Our cell viability experiments showed that high concentrations of caffeine-DHA produced cell toxicity. Nevertheless, taking into consideration its pharmacological activity, caffeine-DHA is a promising drug for targeting A_2A_R in pathologies in which its constitutive activity is altered (i.e., PD). Finally, our approach of attaching DHA to a small drug may be a good model for future drug discovery studies.
